# Results of repeat manipulation under ultrasound-guided cervical nerve root block with corticosteroid and local anaesthetic injection for recurrence of frozen shoulder

**DOI:** 10.1186/s13018-020-02120-8

**Published:** 2020-12-07

**Authors:** Tatsuki Oshiro, Masayoshi Yagi, Kazuki Harada, Kieun Park

**Affiliations:** 1 Yagi Orthopaedic Clinic, 47-1, Zike-machi, Kakogawa-cho, Kakogawa, Hyogo 675-0066 Japan; 2 Paku Pain Clinic, 6-1-20, Gokodori, Chuo-ku, Kobe, Hyogo 651-0087 Japan

**Keywords:** Idiopathic frozen shoulder, MUC, Repeat MUC

## Abstract

**Background:**

This study aimed to evaluate the clinical results of a repeat manipulation under ultrasound-guided cervical nerve root block (MUC) with corticosteroid and local anaesthetic injection for recurrence of idiopathic frozen shoulder after MUC.

**Methods:**

A consecutive series of 42 shoulders in 39 patients with idiopathic frozen shoulder underwent MUC. All patients were assessed according to the American Shoulder Elbow Surgeon (ASES) score and shoulder range of motion (ROM) both before MUC and at 1 year thereafter. If patients continued to have pain and limited ROM at 3 months after the procedure, they were offered a repeat MUC. Such patients were also assessed before the procedure and at 3 months and 1 year thereafter.

**Results:**

The initial MUC was successful in 31 shoulders (single group). Repeat MUC was required in 11 shoulders (repeat group). Patients in the single group showed significant improvement in ROM and ASES score at 1 year after the procedure (*p* < 0.001); similarly, patients in the repeat group had significant improvement in ROM and ASES score at 3 months and 1 year after the procedure (*p* < 0.001). Patients in the repeat group had had significantly more severely limited ROM (*p* < 0.01) and decreased ASES score (*p* < 0.001) before the procedure compared with those in the single group.

**Conclusions:**

A repeat MUC with corticosteroid and local anaesthetic injection is a valuable option before proceeding to surgery for recurrence of idiopathic frozen shoulder. When there is severely limited ROM and decreased ASES score before the MUC, a repeat MUC may be necessary, which would require the patient’s informed consent.

**Trial registration:**

Retrospectively registered

## Background

Idiopathic frozen shoulder is characterised by a limited range of motion (ROM) and shoulder pain [1]. Although the symptoms of the disease are self-limiting [2, 3], over 50% of patients treated non-operatively for idiopathic frozen shoulder still have symptoms and impaired range of motion (ROM) as much as 7 years after initial follow-up [4]. In cases with residual restriction of motion after conservative treatment, surgical intervention including open surgical release, arthroscopic capsular release and manipulation under anaesthesia (MUA) with or without corticosteroid and local anaesthetic injection may be indicated [5–16].

Recently, however, it has been reported that manipulation under ultrasound-guided cervical nerve root block (MUC) has resulted in good clinical results [17, 18]. However, while a limited number of studies have discussed recurrence in patients who had unsuccessful outcomes with MUC, to the best of our knowledge, no other studies have evaluated the clinical outcomes of repeated MUC (repeat MUC).

The purpose of this study was to evaluate the short-term clinical outcomes of a repeat MUC with corticosteroid and local anaesthetic injection performed when the initial results were less than satisfactory or when patients developed recurrent symptoms after their initial MUC. We also looked at whether there were any differences between the single and repeat MUC groups before the procedure.

## Methods

This was a retrospective case-control study, with a single surgeon and a consecutive series of patients who underwent MUC for idiopathic frozen shoulder between April 2013 and August 2019. The study was approved by the institutional review boards of the authors’ affiliated institutions.

### Inclusion and exclusion criteria

The criteria for MUC were as follows: (1) patients with pain and limited active and passive ROM in all three planes (≤ 120° of forward flexion [FF] and abduction, ≤ 30° of external rotation at the side [ER]); (2) patients who did not respond to conservative therapy such as a combination of non-steroidal anti-inflammatory drugs (NSAIDs), intra-articular steroid injections and physiotherapy for at least 3 months.

Exclusion criteria were as follows: (1) rotator cuff tear, (2) osteoarthritis of the shoulder, (3) any fracture involving the shoulder girdle, (4) diabetics with frozen shoulder and (4) failure to attend 1-year follow-up.

### Patient selection

Forty-two patients (45 shoulders) who underwent MUC for frozen shoulder were evaluated. Of these 42 patients (45 shoulders), 3 patients were excluded due to absence of follow-up for at least 1 year, leaving 39 patients (42 shoulders) who were finally included in the study.

### MUC procedure

MUC is performed with the patient in a supine position. The cervical nerve roots (C5/C6) between the anterior and middle scalene muscle are identified using ultrasound. The patient is then injected with 10 ml of anapeine, 10 ml of normal saline and 10 ml of 1% lidocaine. The scapula is stabilised with one hand, while it is recommended that the other hand should hold the proximal portion of the humerus, using a short lever arm to avoid serious complication. The shoulder is manipulated sequentially through a range of abduction, external rotation, forward flexion and cross-body adduction followed by internal rotation flexion. Finally, the arm is extended and rotated internally. After the MUC procedure, 2 mL of 1% lidocaine plus 40 mg of triamcinolone is injected into the joint to prevent postoperative pain.

### Postoperative physiotherapy

All patients attended physiotherapy three times a week for at least 3 months. Such therapy included relaxation of the muscles around the shoulder girdle, gleno-humeral mobilisation and passive capsular and muscle stretching were applied to avoid pain. At home, patients were instructed to follow through with a home flexibility programme once every 4 to 5 h for the first week after the procedure. The programme included five repetitions of passive stretches for flexion utilising a cane.

### Inclusion criteria for repeat MUC

Patients who had a good response after their initial MUC but presented at a later date with recurrent symptoms or those whose initial MUC was less than satisfactory were offered a repeat MUC. Inclusion criteria for the repeat MUC were as follows: (1) patients who did not respond to physiotherapy for at least 3 months after MUC, (2) patients who continued to have pain defined as 5/10 or greater on the visual analogue scale (VAS) for pain and (3) patients who had limited ROM in all three planes (≤ 135° of FF and abduction, ≤ 35° of ER).

### Outcome assessment

All patients were assessed before the procedure and at 1 year after MUC. Outcome measures included ROM of the shoulder (FF, abduction and ER) and the American Shoulder Elbow Surgeons (ASES) score.

The ASES score includes two parameters: pain and daily life activities. The maximum score for pain was 50 points (range, 0 to 10; where 0 = no pain and 10 = severe pain). Also, a maximum of 50 points were assigned for the ability to carry out daily activities. Therefore, the total ASES score was 100 points.

Patients who had a repeat MUC were also assessed before the procedure, at 3 months (i.e., before the repeat MUC) and at one year after the procedure.

### Statistical analysis

We used unpaired *t* test to compare the averages of continuous variables (such as age, duration of symptoms, ROM, ASES score) and chi-square tests to compare the proportion of categorical factors, such as sex, between the groups. The level of significance was set at *p* < 0.05. All analyses were performed with the use of SPSS software for Windows (version 20).

## Results

Of the 39 patients (42 shoulders), 31 patients were women (79%) and 8 patients were men (21%), with a mean age of 53 ± 7 years and a mean follow-up period of 15 months (range 12 to 36 months). Prior to the procedure, the average duration of symptoms was 8.5 months (range 6 to 21 months). There were 3 patients (11%) who had bilateral frozen shoulder at different times. All 39 patients were managed with a supervised physiotherapy programme for at least 3 months after MUC. After completion of the programme, 31 shoulders (74%) showed decreased pain and improved motion (single group). The remaining 11 shoulders (26%) continued to have both loss of motion and pain and required a repeat MUC (repeat group).

### Single group

**Fig 1:**
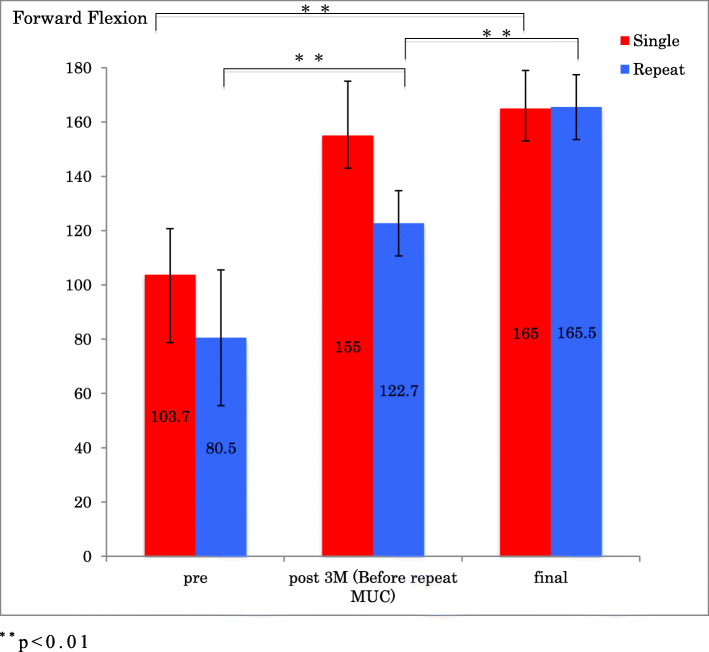
Forward flexion before and after single MUC and repeat MUC. ^**^p < 0.01

**Fig 2:**
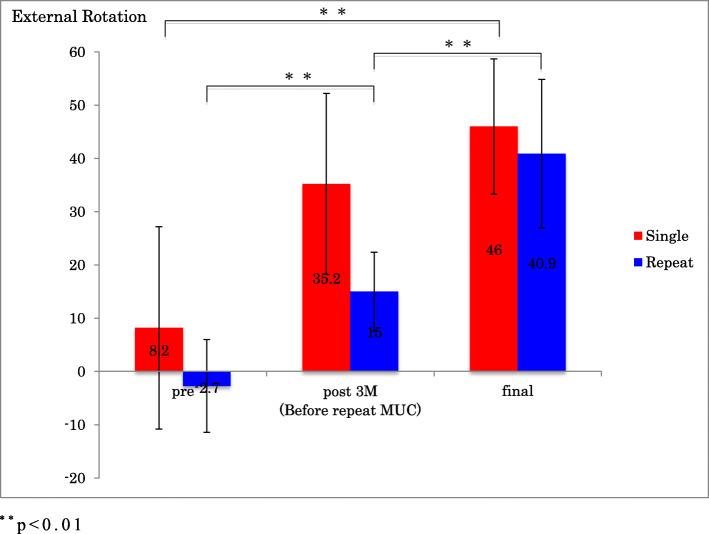
External rotation before and after single MUC and repeat MUC. ^**^p < 0.01

**Fig 3:**
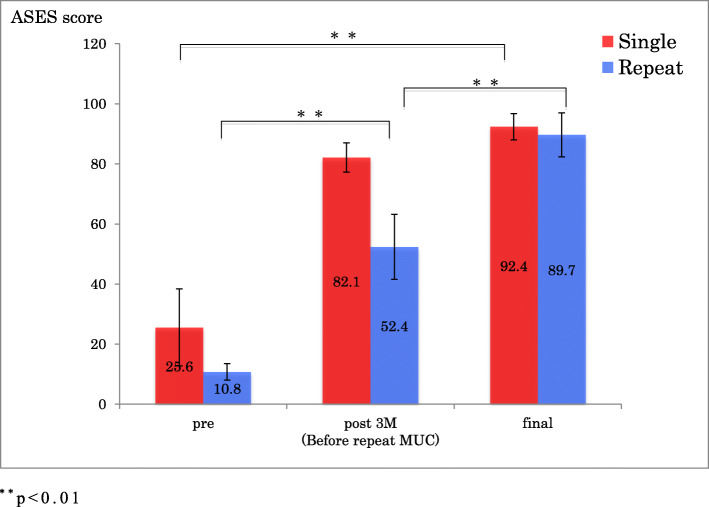
ASES score before and after single MUC and repeat MUC. ^**^p < 0.01

**Fig 4:**
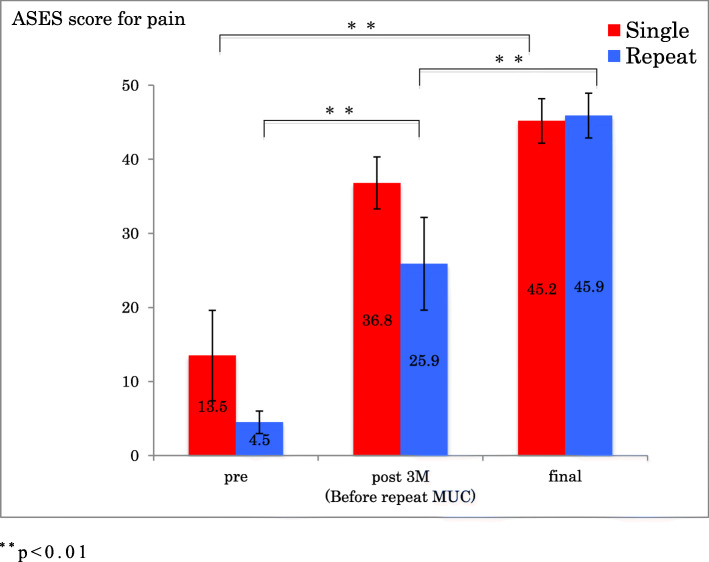
ASES score for pain before and after single MUC and repeat MUC. ^**^p < 0.01

In the single group, there was a statistically significant improvement in ROM of forward flexion, external rotation, ASES scores and ASES score for pain from before the procedure to follow-up 1 year after MUC (*p* < 0.001) (Figs.1, 2, 3 and 4).

### Repeat group

In the repeat group, there was an initial improvement in ROM of forward flexion, external rotation, ASES score and ASES score for pain, which subsequently deteriorated by the time of the repeat MUC, after which ROM, ASES scores and ASES score for pain improved significantly (*p* < 0.001) (Figs.1, 2, 3 and 4).

### Comparison of patient characteristics

The requirement for a repeat MUC did not appear to be related to age, sex or duration of symptoms between the single and repeat groups. However, compared with the single group, the repeat group had revealed a significant severely limited ROM (*p* < 0.01) and a decrease in ASES score before the procedure (*p* < 0.01) (Table 1).

**Table 1:** Patient characteristics

	Single	Repeat	*P*
Mean age	53 ± 7	53 ± 7	NS
Sex (female/male)	22/8	9/1	NS
Duration of symptoms (months)	5.4 ± 2	5.5 ± 2	NS
FF before MUC	103.7 ± 17	80.4 ± 22	0.007
ER before MUC	8.2 ± 16	− 2.7 ± 7	0.005
ASES score before MUC	25.6 ± 12	10.8 ± 2	0.0001

*FF* forward flexion, *ER* external rotation, *MUC* manipulation under ultrasound-guided cervical nerve root block, *ASES* American Shoulder and Elbow Surgeons, *NS* not significant

### Complications

There were no complications, such as fracture or dislocation, and no symptoms of acute rotator cuff tear, neurological or other iatrogenic injuries.

## Discussion

This study confirmed that a repeat MUC with corticosteroid and local anaesthetic injection for recurrence of idiopathic frozen shoulder after MUC results in sustained improvement in ROM and ASES score. Patients who received a repeat MUC showed significantly severely limited ROM and a decreased ASES score before the procedure. There were no complications in our study.

The pathophysiology of frozen shoulder has been thought to be a combination of synovial inflammation, capsular fibrosis and chondrogenesis [19]. Cytokines and growth factors related to fibrosis and inflammation increase in joint capsule from frozen shoulder [19]. Yinghua et al. [20] suggested that neoinnervation and neoangiogenesis in the shoulder capsule are important events in the pathogenesis of frozen shoulder and help explain often-severe pain that patients with frozen shoulder experience.

Two recent studies have reported success with MUC for idiopathic frozen shoulder [17, 18]. Both studies performed MUC with corticosteroid and local anaesthetic injection. Sasanuma et al. [18] performed MUC in 30 shoulders and revealed MR imaging evidence of capsular tear in 29 shoulders (96%). Loew et al. [21] performed arthroscopy in 30 shoulders after closed MUA and reported an 80% incidence of capsular tear in 24 shoulders. These results suggest that MUC procedure may have contributed to joint mobility and pain relief by rupturing the joint capsule which is one of main pathologies of frozen shoulder.

Furthermore, there is suggestion that intra-articular steroid injection may be beneficial in the short term for frozen shoulder but the effect may be limited and not well maintained [19]. Steroid injection is administered to reduce the synovial inflammation to inhibit capsular fibrosis [22]. Therefore, the addition of corticosteroid and local anaesthetic injection may have contributed to reducing inflammation and pain relief.

In a series of reports by Saito et al. [17], the adjusted ASES score at 1-year follow-up was, on average, 93 ± 9 points, which was similar to our results. On the other hand, in our study, 11 of 42 shoulders (26%) developed recurrent moderate pain and limited range of motion after the procedure.

Generally, if patients continue to report significant pain, loss of range of motion and functional impairment after MUA, more aggressive interventions such as open or arthroscopic capsular release or manipulation under general anaesthesia may be indicated and most studies show some benefit [16, 23, 24]. However, such techniques are more invasive and require general anaesthesia and hospitalisation.

To the best of our knowledge, there have been no studies involving the treatment of patients who fail to improve after MUC, nor have there been any published results regarding repeated MUC.

In our study, a total of 11 shoulders (26%) required a repeat MUC if they had recurrence at 3 months after the procedure. We found that ROM (forward flexion, external rotation), ASES scores and ASES score for pain showed significant improvement after the initial and the repeat MUC. In a prospectively collected study, Woods and Loganathan [23] performed MUA in 730 patients with frozen shoulder. A total of 141 patients (17.8%) required a further MUA. This study found that there was an initial improvement in the Oxford Shoulder Score (OSS), which subsequently deteriorated by the time of the further MUA, after which the scores significantly improved, these finding are consistent with our results.

In addition, they reported that in those patients who required a further MUA, the pre-operative ROM was greater than it had been before the initial MUA [23]. In our study, in patients who required repeat MUC, the pre-operative ROM of forward flexion, external rotation, ASES score and ASES score for pain were greater than before the initial MUC. These results suggest that if patients continue to report significant pain and limited range of motion after MUC, repeat MUC leads to improvement not only in range of motion but also in shoulder pain.

In our study, patients in the repeat group displayed significantly more severely limited ROM and worse ASES scores before their initial MUC compared with those in the single group. This is in contrast to the study by Woods and Loganathan [23], who found that there was no significant difference in mean pre-operative ROM between the groups for initial MUA. The exact cause is unclear, though muscle contraction or other connective tissue responses might have been more severe in our repeat group. Furthermore, arthroscopic capsular release allows precise and controlled release of the capsule and ligaments [19]. Closed manipulation, on the other hand, is associated with concerns regarding inadequate release of the articular capsule. The possibility that the articular capsule and ligament were more severe in our repeat group cannot be ruled out.

We recognise there are several limitations in our study. First, this was a retrospective study design. Second, our patient population was smaller than other studies. Third, the follow-up period in this study was quite short term; a longer-term follow-up is necessary to evaluate the efficacy of a repeat MUC.

## Conclusion

In conclusion, patients with idiopathic frozen shoulder treated with MUC had significant improvement in shoulder ROM, pain and shoulder function. If patients continue to have pain and limited ROM after MUC, a repeat MUC with corticosteroid and local anaesthetic injection can be a valuable option before proceeding to surgery. A repeat MUC is likely to be required when there is severely limited ROM and decreased ASES score before the procedure, and this should be explained to the patient and their informed consent obtained.

## Data Availability

Not applicable.

## Abbreviations

MUC: Manipulation under ultrasound-guided cervical nerve root block
ASES: American Shoulder Elbow Surgeon
ROM: Range of motion
MUA: Manipulation under anaesthesia
FF: Forward flexion
ER: External rotation
OSS: Oxford Shoulder Score
